# Robust mutant strain design by pessimistic optimization

**DOI:** 10.1186/s12864-017-4025-7

**Published:** 2017-10-03

**Authors:** Meltem Apaydin, Liang Xu, Bo Zeng, Xiaoning Qian

**Affiliations:** 1 Dept. of Electrical and Computer Engineering, Texas A&M University, College Station, 77843 USA; 2 Dept. of Industrial Engineering, University of Pittsburgh, Pittsburgh, 15260 USA

**Keywords:** Strain optimization, Pessimistic bi-level optimization, Stoichiometric models

## Abstract

**Background:**

Flux Balance Analysis (FBA) based mathematical modeling enables *in silico* prediction of systems behavior for genome-scale metabolic networks. Computational methods have been derived in the FBA framework to solve bi-level optimization for deriving “optimal” mutant microbial strains with targeted biochemical overproduction. The common inherent assumption of these methods is that the surviving mutants will always cooperate with the engineering objective by overproducing the maximum desired biochemicals. However, it has been shown that this optimistic assumption may not be valid in practice.

**Methods:**

We study the validity and robustness of existing bi-level methods for strain optimization under uncertainty and non-cooperative environment. More importantly, we propose new pessimistic optimization formulations: P-ROOM and P-OptKnock, aiming to derive robust mutants with the desired overproduction under two different mutant cell survival models: (1) ROOM assuming mutants have the minimum changes in reaction fluxes from wild-type flux values, and (2) the one considered by OptKnock maximizing the biomass production yield. When optimizing for desired overproduction, our pessimistic formulations derive more robust mutant strains by considering the uncertainty of the cell survival models at the inner level and the cooperation between the outer- and inner-level decision makers. For both P-ROOM and P-OptKnock, by converting multi-level formulations into single-level Mixed Integer Programming (MIP) problems based on the strong duality theorem, we can derive exact optimal solutions that are highly scalable with large networks.

**Results:**

Our robust formulations P-ROOM and P-OptKnock are tested with a small *E. coli* core metabolic network and a large-scale *E. coli* iAF1260 network. We demonstrate that the original bi-level formulations (ROOM and OptKnock) derive mutants that may not achieve the predicted overproduction under uncertainty and non-cooperative environment. The knockouts obtained by the proposed pessimistic formulations yield higher chemical production rates than those by the optimistic formulations. Moreover, with higher uncertainty levels, both cellular models under pessimistic approaches produce the same mutant strains.

**Conclusions:**

In this paper, we propose a new pessimistic optimization framework for mutant strain design. Our pessimistic strain optimization methods produce more robust solutions regardless of the inner-level mutant survival models, which is desired as the models for cell survival are often approximate to real-world systems. Such robust and reliable knockout strategies obtained by the pessimistic formulations would provide confidence for in-vivo experimental design of microbial mutants of interest.

## Introduction

Whole-genome high-throughput profiling techniques have enabled the systematic study of biological systems at the genome scale [1, 2]. In particular, systems models and computational methods for analyzing and controlling genome-scale metabolic networks have greatly advanced the field of metabolic engineering [3]. With the better understanding of the systems behavior of microbial metabolism, metabolic engineering based on the metabolic network models can help predict metabolic phenotypes [4, 5] and derive engineering strategies for strain design by manipulating the native microbial pathways to produce chemicals of commercial and biomedical benefits [6–11].

Mathematical modeling of metabolism often focuses on steady-state behavior, especially when long-term metabolic dynamics is of interest, as accurate reconstruction of genome-scale kinetic models is challenging when considering the large model space and parameter uncertainties. Constraint-based approaches based on the reaction stochiometry, notably Flux Balance Analysis (FBA) by Linear Programming (LP) formulations, study genome-scale dynamics by mass-balance equations at steady states to understand and predict macro-level microbial behavior in the presence of perturbation, for example caused by mutations or environmental changes [12–15]. By adding thermodynamic and flux capacity constraints, FBA often models steady-state behavior by assuming that the cell growth flux needs to be maximized based on biomass composition [16].

Many computational approaches have been proposed in this computational framework for *in silico* prediction of potentially feasible metabolic phenotypes and evaluation of theoretical limits of metabolic performance after knocking out certain genes or reactions for strain design [14, 16]. Metabolic engineering with such computationally derived knockout strategies has shown to be effective to achieve overproduction of biochemicals of interest [17]. Those strain optimization methods are generally modeled as bi-level optimization problems that seek for maximum overproduction of a desired biochemical at the outer level under the condition that mutant cells are still alive, modeled as the inner-level optimization problem. For example, OptKnock [17], ROOM (Regulatory On/Off Minimization) [18], and MOMAKnock [19] all fall under this category with different mutant cell survival models at the inner level detailed in the “Background” section.

These existing bi-level formulations for microbial strain design have inherent assumptions that nature will always cooperate with the human desire to select the mutants that serve the outer-level engineering objective the best, namely they assume that a cooperative environment exists between the outer-level (human) and inner-level (microbial strain) decision makers. However, in practice, surviving mutant strains may not always fit the best with the engineering goal. In addition, the model assumptions for the cell survival at the inner level are often the approximations to the real-world scenarios, which may result in the derived knockout strategies not overproducing the predicted amount of desirable biochemicals. Therefore, the robustness and viability of these predicted knockout strategies may need to be re-evaluated. For example, if the perturbed strains do not cooperate with the knockout implementer, they may do very opposite to the engineering objective and these optimistic bi-level strategies may not work in practice. In [20], we have shown that OptKnock-derived knockout strategies may produce the desired chemicals at levels much lower than the expected ones, when the aforementioned two assumptions are relaxed.

**Fig. 1:**
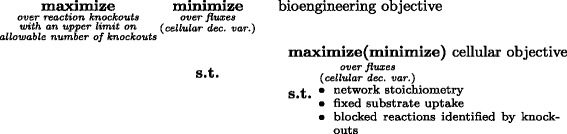
The pessimistic bi-level problem structure for knockout identification. The inner-most level problem defines the optimization of cell survival based on a specific cellular objective (e.g., maximization of biomass growth in P-OptKnock and minimization of flux changes in P-ROOM). The outer level maximizes the worst-case scenario for Succinate production

In this paper, we innovate a computational framework to predict robust knockout strategies for overproduction of desirable chemicals with different inner-level mutant cell survival models. Specifically, we develop a pessimistic optimization formulation [21] that considers the uncertainty of the cell survival models at the inner level and the cooperation between the outer- and inner-level optimization decisions. Figure 1 illustrates our pessimistic bi-level optimization framework for identifying the knockouts to have the maximum overproduction of a desired chemical as a bioengineering objective under the worst-case or least-favorable scenario. The inner-level cellular model has a competing objective function to maintain the cell survival. According to the cellular model, different objective functions can be chosen. We study such pessimistic formulations with both the cell survival criteria of (1) minimization of significant flux changes with respect to wild-type flux values as in ROOM [18]; and (2) maximization of biomass growth as in OptKnock [17]. Based on the corresponding pessimistic formulations, which we call P-ROOM and P-OptKnock respectively, we will optimize the desired overproduction in the least favorable situation (i.e., the non-cooperative situation) and investigate the sensitivity of resulting knockouts with respect to cell response and uncertainty introduced by the inner-level cell survival models. Since the inner-level cellular model is linear given the outer-level binary decision variables, we can employ the LP duality theory and convert the pessimistic bi-level optimization problem to a single-level MIP problem by applying two dual transformations as described in Methods. Experimental results on both a core *E. coli* metabolic network and a genome-scale iAF1260 network [22] show that our pessimistic formulations can generate more robust knockout strategies compared to the existing bi-level optimization methods for strain design. Regardless of the choice of inner-level surrogate functions for cell survival, P-ROOM and P-OptKnock produce consistent and stable knockout strategies for the targeted biochemical overproduction as the formulations are specifically designed to take care of the model uncertainty.

## Background

Before we present pessimistic optimization formulations to derive robust knockout strategies for strain design, we briefly summarize the background and important mathematical notations for FBA and the bi-level optimization models for deriving knockouts, including OptKnock [17] and ROOM [18].

### FBA

FBA is an LP problem for the analysis of stoichiometric-based metabolic network models of involved reactions at the genome scale. A linear objective function is minimized or maximized subject to mass-balance, thermodynamic and capacity constraints, with respect to reaction fluxes in a vector form **v**. The LP formulation of FBA can be expressed as:

$$\begin{aligned} & \text{max(min)}_{\mathbf{v}} \quad \mathbf{c}^{T}\mathbf{v} \\ & \text{s.t.} \quad \mathbf{Sv}=\boldsymbol{0}, \quad \mathbf{v}_{min}\leq \mathbf{v} \leq \mathbf{v}_{max}. \end{aligned} $$

Under the steady-state assumption, mass-balance constraints constitute a system of linear equations where the weighted sum of fluxes, based on stoichiometric coefficients given in a matrix form **S**, is 0. Thermodynamic and capacity constraints are defined as lower and upper bounds on reaction fluxes. In the FBA framework, maximization of biomass growth is often adopted as the objective function for modeling cell survival, where **c** becomes a vector with all values of 1 for the reactions corresponding to the biomass formation.

### OptKnock

FBA enables efficient computational design of beneficial genetically engineered microbial strains. The pioneering work by the authors of [17], OptKnock, searches for potential genetic perturbations (e.g., gene or reaction knockouts) for redirection of metabolic flux to overproduce desired biochemicals and maintain cellular growth. This bi-level optimization problem captures the engineering objective at the outer level (e.g., to maximize the overproduction) while the inner-level problem models a cellular fitness objective (e.g., maximization of biomass growth). The mathematical programming formulation of OptKnock can be expressed as follows:

1 $$ \begin{aligned} & \underset{z_{j} \in \left\lbrace 0, 1\right\rbrace,\forall j \in J}{\text{max}} \quad v_{chemical} \\ & \text{s.t.} \quad \underset{v_{j} \in \mathbb{R},\forall j \in J}{\text{max}} \quad v_{biom}\\ & \qquad \text{s.t.} \quad \sum_{j \in J} S_{ij}v_{j} = 0, \quad \forall {i \in I}\\ & \qquad \qquad \quad v_{glc} = v_{glc\_uptake}, \quad v_{biom} \geqslant v_{biomass}^{target}\\ & \qquad \qquad \quad v_{j}^{min}z_{j} \leqslant v_{j} \leqslant v_{j}^{max}z_{j}, \quad \forall {j \in J}\\ & \qquad \sum_{j\in J}(1- z_{j}) \leqslant K,\\ \end{aligned}  $$

where *I* and *J* are the sets of metabolites and reactions, respectively. In this model, *v*_*j*_ represents the flux of reaction *j* in *J*. Each element of the matrix **S** is the stoichiometric coefficient *S*_*ij*_ of metabolite *i* in reaction *j*. The outer-level binary decision variable *z*_*j*_ is 1 if the corresponding reaction flux *v*_*j*_ is active and 0 otherwise. The overproduction of a chemical of interest is maximized at the outer level allowing *K* possible knockout reactions. The inner-level cell survival model is based on steady-state analysis with FBA constraints. Depending on the availability of nutrients or the maximal fluxes that can be supported by enzymatic pathways [13], $v_{j}^{min}$ and $v_{j}^{max}$ are the lowest and highest possible reaction fluxes for the reaction *j*, respectively. The glucose consumption rate *v*_*glc*_ is often set to a fixed value as $v_{glc\_uptake}$, and $v_{biomass}^{target}$ is the minimum level of biomass production. As the biomass growth is a linear objective function of metabolic reaction fluxes, strong duality of the inner-level optimization helps to convert the original bi-level optimization problem into a Mixed Integer Linear Programming (MILP) problem, which can be solved efficiently for large-scale metabolic networks [17, 23].

### ROOM

Observed experimental flux values reported in [24] showed that maximizing biomass reaction flux as a surrogate function for the most probable physiological state of the metabolic model is indeed effective for wild-type strains as they have been exposed to long-term evolutionary pressure. However, it may not be valid for the engineered mutants without such a long-term progress. Therefore, it calls for other realistic cell survival models for mutants. ROOM [18] is one of such models, in which binary variables *y*_*j*_’s are introduced to capture significant (up/down regulated) or insignificant reaction flux changes. Instead of maximizing the biomass growth, ROOM [18] aims to minimize the number of significant flux changes. Relaxing the binary variables *y*_*j*_’s in the original MILP formulation leads to the LP variant of ROOM:

2 $$ \begin{aligned} & \underset{z_{j} \in \left\lbrace 0, 1\right\rbrace,\forall j \in J}{\text{max}} \quad v_{chemical} \\ & \text{s.t.} \quad \underset{v_{j} \in \mathbb{R},y_{j} \in \mathbb{R},\forall j \in J}{\text{min}} \quad \sum_{j\in J} y_{j}\\ & \qquad \text{s.t.} \quad \sum_{j\in J} S_{ij}v_{j} = 0, \forall {i} \in I\\ & \qquad \qquad \quad v_{glc} = v_{glc\_uptake}, \quad v_{biom} \geqslant v_{biomass}^{target}\\ & \qquad \qquad \quad v_{j}^{min}z_{j} \leqslant v_{j} \leqslant v_{j}^{max}z_{j}, \forall {j} \in J\\ & \qquad \qquad \quad v_{j}-y_{j}(v_{j}^{max}-w_{j}) \leq w_{j}, \forall {j} \in J\\ & \qquad \qquad \quad v_{j}-y_{j}(v_{j}^{min}-w_{j}) \geq w_{j}, \forall {j} \in J\\ & \qquad \qquad \quad 0 \leq y_{j} \leq 1, \forall {j}\in J\\ & \qquad \sum_{j\in J}(1- z_{j}) \leqslant K,\\ \end{aligned}  $$

in which *w*_*j*_ is the wild-type flux values that can be solved by FBA. In this bi-level optimization model (2), the inner-level cellular objective is to minimize the flux changes. The inequalities with *y*_*j*_’s constrain the flux values not to deviate from the wild-type flux values. We again can employ the strong duality condition to convert the bi-level optimization problem into a MILP to solve (2).

## Methods

The above optimistic bi-level optimization methods effectively model the interacting objectives of the outer-level knockout implementer and the inner-level microbial cells. However, the effectiveness of these optimistic formulations depends on the inherent assumption that the outer-level engineering objective and inner-level cellular fitness function behave cooperatively by selecting the inner-level solutions in favor of the outer-level optimization problem. In practice, when the inner-level problem either does not faithfully reflect cellular fitness objectives or has non-unique solutions, there is no guarantee that the cell response will be in cooperation with the engineering objective to maximize the desirable biochemical product formation as we recently found in [20]. The main questions that we would like to address here are:

1. *How robust are the derived knockout strategies based on these optimistic bi-level optimization formulations?*
2. *Does the robustness depend on how the inner-level optimization problem approximates mutant cell objectives?*
3. *Can we formulate new optimization problems that derive more robust solutions?*

We answer the first two questions by allowing the inner-level problem to take non-optimistic solutions as we have done in [20] to evaluate the knockout strategies derived by OptKnock and ROOM. More importantly, we propose a novel pessimistic bi-level optimization framework in this paper to derive robust knockout strategies, which consider the uncertainty introduced by the inner-level models from the aforementioned non-cooperative and non-unique issues.

We now present our pessimistic optimization formulations for deriving robust mutant strains and provide the detailed derivation of the optimization solutions to our pessimistic models, in which we maximize the desired overproduction for the worst-case scenario due to uncertainty and non-cooperative environment with different inner-level mutant cell survival models.

### Pessimistic bi-level optimization

As discussed, due to the inner-level model uncertainty and non-cooperative issues, the existing optimistic bi-level strain optimization methods may not be able to produce reliable results when there exist non-unique solutions to the inner-level problem. In order to overcome this limitation, we propose a pessimistic optimization framework to optimize for the worst-case scenario under uncertainty and non-cooperative environment. The mathematical programming formulation of pessimistic bi-level optimization can be represented in the following general form [21]:

3 $$  {}\begin{aligned} & \underset{\mathbf{x}\in\mathbb{X}}{\text{max}} \quad \underset{\mathbf{y}\in Y(\mathbf{x})}{\text{min}} \quad \mathbf{F}(\mathbf{x},\mathbf{y})\\ & \text{s.t.} \quad \mathbf{G}(\mathbf{x})\leq 0 \\ & \quad \quad~ Y(\mathbf{x}) = \text{arg min(max)}_{\mathbf{y}} \lbrace \mathbf{f}(\mathbf{x},\mathbf{y}) : \mathbf{g}(\mathbf{x},\mathbf{y})\leq0, \mathbf{y}\in\mathbb{Y}\rbrace, \end{aligned}  $$

where **x** and **y** are the outer-level and inner-level decision variables with the corresponding feasible sets $\mathbb {X}$ and $\mathbb {Y}$. In the above formulation, **F** represents the outer-level objective function, $\mathbf {F}:\mathbb {X}\times \mathbb {Y}\rightarrow \mathbb {R}$, and **f** represents inner-level objective function, $\mathbf {F}:\mathbb {X}\times \mathbb {Y}\rightarrow \mathbb {R}$. **G** and **g** represent the inequality constraint functions at the outer and inner levels, respectively. *Y*(**x**) denotes the set of optimal solutions to the inner-level problem for a given **x**, which may contain multiple elements. Solving such a pessimistic bi-level optimization problem is computationally very difficult. Even a linear optimistic bi-level problem, where both the outer-level and the inner-level problems are linear programs, is theoretically NP-hard [25]. In our study, to solve the more difficult pessimistic bi-level optimization problem, we first employ the LP duality theory based on the fact that the inner-most level problem is a linear program. It converts the pessimistic bi-level model into a standard bi-level problem, which enables us to fully make use of existing bi-level optimization algorithms. For example, we transform the standard bi-level model into a single level mixed integer programming (MIP) problem using the strong duality theory, which can be solved through the commercial MIP solver CPLEX. As those operations are rather simple, we make a challenging pessimistic bi-level problem practically solvable even for large-scale cases.

We mention that for P-ROOM (4), there are |*J*| binary variables in the outer level, 2|*J*| continuous variables and *O*(|*I*|+5|*J*|) constraints in the inner level. By taking the dual of the inner-most problem, we convert (4) into a standard bi-level problem (7), whose inner-level problem has *O*(|*I*|+7|*J*|) variables and *O*(|*I*|+7|*J*|) constraints. Comparing to the traditional bi-level ROOM model (2), which has the identical outer-level problem, and 2|*J*| continuous variables and *O*(|*I*|+5|*J*|) constraints in the inner level, (7) does not have a much larger or more complicated structure. Hence, it can be expected that the computational complexity of (7) will not be drastically more than that of the traditional ROOM model. Moreover, our numerical study shows that the single level MIP reformulation of (7) for a larger-scale network can be computed by CPLEX in a reasonable time, which indicates the efficiency of our proposed solution strategy.

Inspired by this pessimistic optimization framework, we propose pessimistic models for mutant strain optimization under model uncertainty. We note that the notations appearing in the following pessimistic formulations have the same definitions as in the “Background” section.

### Pessimistic strain optimization I: P-ROOM

In the following, we propose the pessimistic formulation based on the original ROOM model (2) in the same *minimax* flavor as in (3):

4 $$ {\begin{aligned} &\underset{z_{j},\forall j\in J}{\text{max}}~\underset{v_{j},y_{j}\in Y(z_{j}),\forall j\in J}{\text{min}}~v_{chemical} \\ &\text{s.t.}~\sum_{j\in J}\left(1- z_{j}\right) \leqslant K,~z_{j} \in \left\lbrace 0, 1\right\rbrace, \forall j\in J \\ &\quad Y(z_{j})=\text{argmin}_{v_{j},y_{j}} \left\lbrace \sum_{j\in J} y_{j} : \sum_{j\in J} S_{ij}v_{j} \,=\, 0, \forall i\in I, v_{glc} \,=\, v_{glc\_uptake}, \right.\\ &\qquad v_{biom}\!\geqslant\! v_{biomass}^{target},~v_{j}^{min}z_{j} \!\leqslant\!\! v_{j} \leqslant\! v_{j}^{max}z_{j},~v_{j}\,-\,y_{j}\left(v_{j}^{max}-w_{j}\right) \leq w_{j},\\ &\qquad \qquad~\left.v_{j}-y_{j}\left(v_{j}^{min}-w_{j}\right) \geq w_{j},~0\leq y_{j} \leq1, \forall j\in J {\vphantom{\sum_{j\in J}}}\right\rbrace. \end{aligned}}  $$

Originally introduced in [21], the *ε*-approximation extension of the above pessimistic formulation is flexible when model uncertainty needs to be considered for bi-level decision making. Specifically, the *ε*-approximation of the pessimistic problem is to allow a proportional gap of *ε* for the inner-level objective function value from the optimal value that the actual knockout solutions would take. When *ε*=0, we have the original pessimistic formulations assuming that the inner-level models are faithful, but inner-level decision variables may act against outer-level engineering objectives. Higher *ε* values reflect that the inner-level mutant strains have a higher tolerance level for inner-level model uncertainty when a non-cooperative decision is taken against the outer-level engineering objectives. Such an *ε*-approximation of the pessimistic problem can be written as follows:

5 $$ \begin{aligned} &\underset{z_{j}:z_{j}\in \left\lbrace 0,1\right\rbrace,\forall j\in J, \sum_{j\in J}(1- z_{j}) \leqslant K}{\text{max}}~\underset{v_{j},y_{j},\forall j \in J}{\text{min}}~v_{chemical} \\ &\qquad \quad~\text{s.t.}~\sum_{j\in J} S_{ij}v_{j} = 0, \forall i\in I, v_{glc} = v_{glc\_uptake}, v_{biom}\geqslant v_{biom}^{target}\\ &\qquad \qquad\quad~v_{j}^{min}z_{j} \!\!\leqslant\! \!v_{j} \leqslant v_{j}^{max}z_{j},~v_{j}-y_{j}\left(v_{j}^{max}-w_{j}\right) \leq w_{j}, \forall j\in J \\ &\qquad \qquad\quad~v_{j}-y_{j}\left(v_{j}^{min}-w_{j}\right) \geq w_{j},~0\leq y_{j} \leq1, \forall j\in J \\ &\qquad \qquad\quad~\sum_{j\in J}{y_{j}}\leq \varphi(\mathbf{v}^{*},\mathbf{y}^{*})(1+\epsilon), \end{aligned}  $$

where *φ*(**v** ^∗^,**y** ^∗^) is the inner-level function value changing with respect to the inner-level decision variables **v** ^∗^ and **y** ^∗^. As the inner-level problem is LP, we can convert the problem into its dual representation by the strong duality theorem as in [17, 26], which is:

6 $$ \begin{aligned} &\underset{glc,u_{i},\forall i\in I,\mu,a_{j},\forall j\in J} {\text{max}}~glc. v_{glc\_uptake} + \mu_{biom} v_{biomass}^{target} + \sum_{j\in J} v_{j}^{min} \mu_{min,j}z_{j} -\\ & \quad \quad\sum_{j\in J}v_{j}^{max} \mu_{max,j}z_{j} - \sum_{j\in J}\mu_{max2,j} w_{j} + \sum_{j\in J}\mu_{min2,j} w_{j} - \sum_{j\in J}a_{j} \\ & \text{s.t.}~\sum_{i\in I}S_{i,glc}u_{i} + glc + \mu_{min,glc} \,-\,\mu_{max,glc} - \mu_{max2,glc} + \mu_{min2,glc} = 0\\ & \quad~~\sum_{i\in I}S_{i,biom}u_{i} + \mu_{biom} + \mu_{min,biom} -\mu_{max,biom} - \mu_{max2,biom}\\ &\qquad\quad+ \mu_{min2,biom} = 0\\ & \quad~~\sum_{i\in I}S_{i,j}u_{i} + \mu_{min,j} -\mu_{max,j} - \mu_{max2,j} + \mu_{min2,j} = 0, \forall {j}\in J, j\\ &\qquad\quad\neq \text{biom, glc}\\ & \quad~~\mu_{max2,j}\left(v_{j}^{max}-w_{j}\right) - \mu_{min2,j}\left(v_{j}^{min}-w_{j}\right) - a_{j} \leq 1, \forall {j}\in J\\ & \quad~~glc,u_{i}\in\mathbb{R}, \forall {i}\in I \quad \mu_{biom},\mu_{min,j},\mu_{max,j},\mu_{max2,j}, \mu_{min2,j}, a_{j}\\ &\qquad\quad\geq0, \forall {j}\in J \end{aligned}  $$

where *u*_*i*_ denotes the dual variable associated with the mass-balance constraint, $\sum _{j} S_{ij}v_{j} = 0$ for metabolite *i*, *glc* is the dual variable associated with the glucose uptake constraint, *μ*_*biom*_ is the dual variable associated with the minimum biomass threshold constraint, and *μ*_*min*,*j*_ and *μ*_*max*,*j*_ are the dual variables for the two directions of the inequality constraint $v_{j}^{min}z_{j} \leqslant v_{j} \leqslant v_{j}^{max}z_{j}$ for each reaction *j*. Finally, *μ*_*min*2,*j*_ and *μ*_*max*2,*j*_ are the dual variables associated with the constraints $v_{j}-y_{j}\left (v_{j}^{min}-w_{j}\right) \geq w_{j}$ and $v_{j}-y_{j}\left (v_{j}^{max}-w_{j}\right)\leq w_{j}$, denoting the flux changes with respect to wild-type flux values, respectively, and *a*_*j*_ is the dual variable associated with the upper bound constraint on *y*_*j*_. Note that the constraint $v_{j}^{min}z_{j} \leqslant v_{j} \leqslant v_{j}^{max}z_{j}$ links the outer-most level binary decision variables *z*_*j*_ and inner-level decision variables *v*_*j*_.

By aggregating the inner-level constraints to the outer level with those introduced by its dual in (6) with the adopted *ε*-approximation, the final pessimistic formulation P-ROOM aims to solve the following max-min problem;

7 $$ \begin{aligned} & \underset{z_{j}:z_{j}\in \left\lbrace 0, 1\right\rbrace,\forall j\in J,\sum_{j\in J}(1- z_{j}) \leqslant K}{\text{max}} \quad \underset{v_{j}, y_{j},glc, u_{i}, a_{j}, \mu,\forall i\in I,\forall j\in J}{\text{min}} \quad v_{chemical}\\[-2pt] & \qquad \quad \text{s.t.} \quad \sum_{j\in J} S_{ij}v_{j} = 0, \forall {i}\in I\\[-2pt] & \qquad \qquad \quad v_{glc} = v_{glc\_uptake}, v_{biom} \geqslant v_{biomass}^{target},v_{j}^{min}z_{j} \leqslant v_{j}\\[-2pt] & \qquad \qquad \quad \quad\ \leqslant v_{j}^{max}z_{j}, \forall j\in J\\[-2pt] & \qquad\qquad \quad v_{j}-y_{j}\left(v_{j}^{max}-w_{j}\right) \leq w_{j}, v_{j}-y_{j}\left(v_{j}^{min}-w_{j}\right) \geq w_{j}, 0\\[-2pt] & \qquad \qquad \quad \quad\ \leq y_{j}\leq1, \forall {j}\in J\\[-2pt] & \qquad \qquad \quad \left(glc. v_{glc\_uptake} + \mu_{biom} v_{biomass}^{target} + \sum_{j\in J} v_{j}^{min} \mu_{min,j}z_{j}\right.\\[-2pt] & \qquad \qquad \qquad~-\sum_{j\in J}v_{j}^{max} \mu_{max,j}z_{j}\\[-2pt] & \left.\qquad \qquad \qquad~-\sum_{j\in J}\mu_{max2,j} w_{j} + \sum_{j\in J}\mu_{min2,j} w_{j} - \sum_{j\in J}a_{j} \right)(1+\epsilon) \\ & \qquad \qquad \qquad~- \sum_{j\in J}y_{j} \geq 0\\ & \qquad \qquad \quad \sum_{i\in I}S_{i,glc}u_{i} + glc + \mu_{min,glc} -\mu_{max,glc} - \mu_{max2,glc}\\ &\qquad \qquad \quad \quad + \mu_{min2,glc} = 0 \\ &\qquad \qquad \quad \sum_{i\in I}S_{i,biom}u_{i} + \mu_{biom} + \mu_{min,biom} -\mu_{max,biom}\\ &\qquad \qquad \quad \quad- \mu_{max2,biom} + \mu_{min2,biom} = 0\\ & \qquad \qquad \quad \sum_{i\in I}S_{i,j}u_{i} + \mu_{min,j} -\mu_{max,j} - \mu_{max2,j} + \mu_{min2,j}\\ &\qquad \qquad \quad \quad= 0, \forall j\in J, j\neq \text{biom, glc}\\ & \qquad \qquad \quad \mu_{max2,j}\left(v_{j}^{max} \!\,-\, \!w_{j}\right) \!\!- \! \mu_{min2,j}\left(v_{j}^{min} \!- \!w_{j}\right) \,-\, a_{j} \leq 1, \forall j\in J\\ & \qquad \qquad \quad v_{j}, glc,u_{i} \in \mathbb{R},\forall j\in J, \forall i\in I, \\ & \qquad \qquad \quad \mu_{biom},\mu_{min,j},\mu_{max,j},\mu_{max2,j}, \mu_{min2,j}, a_{j},y_{j}\geq 0,\forall j\in J \end{aligned}  $$

In order to solve the max-min problem above, we use the strong duality theorem again to transform the minimization problem into its dual maximization representation, resulting in its equivalent single-level MIP as:

8 $$ \begin{aligned} & \underset{z_{j},\gamma, p_{j},q_{j},x,c_{j},t,s_{j},r_{j}}{\text{max}} \quad \gamma_{glc}v_{glc\_uptake} + \gamma_{biom}v_{biomass}^{target} + \sum_{j\in J}v_{j}^{min}x_{min,j}z_{j} - \\ &\qquad \qquad \quad \sum_{j\in J}v_{j}^{max}x_{max,j}z_{j} - \sum_{j\in J}x_{max2,j}w_{j} + \sum_{j\in J}x_{min2,j}w_{j}\\[-2pt] &\qquad\qquad\qquad-\sum_{j\in J}c_{j} - \sum_{j\in J}q_{j}\\[-2pt] & \text{s.t.}\qquad\sum_{j\in J}(1- z_{j}) \leqslant K, \\[-2pt] & \quad \qquad\sum_{j\in J}S_{i,j} s_{j} = 0, \forall i\in I,~s_{glc} = v_{glc\_uptake} \\[-2pt] & \quad \qquad s_{biom} \geqslant v_{biomass}^{target},~v_{j}^{min}z_{j} \leqslant s_{j} \leqslant v_{j}^{max}z_{j}, \forall j\in J \\[-2pt] & \quad \qquad s_{j}\,-\,r_{j}\left(v_{j}^{max}\,-\,w_{j}\right)\! \!\leq\! w_{j}, ~s_{j}\!\,-\,r_{j}\left(v_{j}^{min}\,-\,w_{j}\right)\! \!\geq\! w_{j},~0\!\!\leq\!\! r_{j} \!\leq\! 1, \forall {j}\in J\\[-2pt] & \quad \qquad \sum_{i\in I}S_{i,chem}\gamma_{i} + x_{min,chem} - x_{max,chem} - x_{max2,chem}\\[-2pt] & \quad \qquad\quad + x_{min2,chem} = 1\\[-2pt] & \quad \qquad \sum_{i\in I}S_{i,glc}\gamma_{i} + \gamma_{glc} + x_{min,glc} - x_{max,glc} - x_{max2,glc}\\[-2pt] & \quad \qquad \quad + x_{min2,glc} = 0\\[-2pt] & \quad \qquad \sum_{i\in I}S_{i,biom}\gamma_{i} + \gamma_{biom} + x_{min,biom} - x_{max,biom} - x_{max2,biom}\\[-2pt] & \quad \qquad \quad + x_{min2,biom} = 0\\[-2pt] & \quad \qquad \sum_{i\in I}S_{i,j}\gamma_{i} + x_{min,j} -x_{max,j} - x_{max2,j} + x_{min2,j} = 0, \forall {j}\in J, j\\[-2pt] & \quad \qquad \quad \neq \text{biom,glc,chem} \\[-2pt] & \quad \qquad x_{max2,j}\left(v_{j}^{max}\,-\,w_{j}\right) \,-\, x_{min2,j}\left(v_{j}^{min}-w_{j}\right) - c_{j} - t \leq 0, \forall {j}\in J\\[-2pt] & \quad \qquad \sum_{j\in J} S_{ij}p_{j} = 0, \forall {i}\in I \\[-2pt] & \quad \qquad (1+\epsilon)v_{glc\_uptake}t + p_{glc} = 0 \\[-2pt] & \quad \qquad -v_{j}^{max}(1+\epsilon)tz_{j} - p_{j} \leq 0, \forall j\in J \\[-2pt] & \quad \qquad v_{j}^{min}(1+\epsilon)tz_{j} + p_{j} \leq 0, \forall j \in J\\[-2pt] & \quad \qquad (1+\epsilon)v_{biomass}^{target}t + p_{biom} \leq 0 \\[-2pt] & \quad \qquad -w_{j}(1+\epsilon)t - p_{j} - \left(v_{j}^{max}-w_{j}\right)q_{j} \leq 0, \forall j\in J \\[-2pt] & \quad \qquad w_{j}(1+\epsilon)t + p_{j} + \left(v_{j}^{min}-w_{j}\right)q_{j} \leq 0, \forall j\in J \\[-2pt] & \quad \qquad q_{j} - (1 + \epsilon)t \leq 0, \forall j\in J\\[-2pt] & \quad \qquad s_{j}, \gamma_{glc}, \gamma_{i}, p_{j} \in \mathbb{R},\forall i\in I,\forall j\in J\\[-2pt] & \quad \qquad\gamma_{biom}, x_{min,j}, x_{max,j}, x_{max2,j}, x_{min2,j}, c_{j}, t, q_{j}, r_{j} \geq 0, z_{j} \\[-2pt] & \quad \qquad \quad \in\lbrace0,1\rbrace, \forall j\in J. \end{aligned}  $$

Since some knockout strategies *z*_*j*_ may not have corresponding feasible solutions to the inner problem of (7), causing the corresponding dual solutions to be unbounded, new continuous decision variables *s*_*j*_ and *r*_*j*_ are added here in order to enforce the feasibility of the inner primal problem of (7) to avoid such degenerated cases. The bilinear terms in the objective function and the constraints in (8) can be further linearized by using the commonly adopted big-M method. This single-level MILP problem can be solved efficiently for large-scale problems by available solvers.

### Pessimistic strain optimization II: P-OptKnock

In OptKnock [17], the inner-level mutant survival model is approximated by the maximum biomass growth condition. In order to optimize for the overproduction of the target biochemicals in worse-case scenario, we can similarly write the corresponding pessimistic formulation as what we have derived for P-ROOM:

9 $$ \begin{aligned} & \underset{z_{j},\forall j\in J}{\text{max}} \quad \underset{v_{j}\in Y(z_{j})}{\text{min}} \quad v_{chemical} \\ & \text{s.t.} \qquad \sum_{j\in J}(1- z_{j}) \leqslant K \\ & \quad \qquad z_{j} \in \left\lbrace 0, 1\right\rbrace, \forall j\in J\\ & \quad \qquad Y(z_{j})=\text{argmax} \left\lbrace v_{biom}: \sum_{j\in J} S_{ij}v_{j} = 0, \forall {i} \in I,\right. \\ &\qquad \qquad \qquad v_{glc} = v_{glc\_uptake},\\ & \qquad \qquad \qquad \left.{\vphantom{\sum_{j\in J}}}v_{biom} \geqslant v_{biomass}^{target}, \quad v_{j}^{min}z_{j} \leqslant v_{j} \leqslant v_{j}^{max}z_{j}, \forall {j}\in J \right\rbrace. \end{aligned}  $$

By adopting the same *ε*-approximation to the pessimistic problem in (9) to incorporate the inner-level model uncertainty, we have the P-OptKnock formulation as:

10 $$ {}\begin{aligned} &\underset{z_{j}: z_{j} \in \left\lbrace 0,1\right\rbrace,\forall j\in J,\sum_{j\in J}(1- z_{j}) \leqslant K}{\text{max}}~\underset{v_{j}, \forall j\in J}{\text{min}}~v_{chemical} \\ &\qquad \quad~\text{s.t.}~\sum_{j\in J} S_{ij}v_{j} = 0, \forall i\in I, v_{glc} = v_{glc\_uptake}, \\ &\qquad \qquad \quad\,\, v_{biom}\geqslant v_{biomass}^{target}\\ &\qquad \qquad\quad~v_{j}^{min}z_{j} \leqslant v_{j} \leqslant v_{j}^{max}z_{j}, \forall j\in J \\ &\qquad \qquad\quad~v_{biom}\geq \varphi(\mathbf{v}^{*})(1-\epsilon), \end{aligned}  $$

where *φ*(**v** ^∗^) is the inner-most level function value of a given inner-level decision variable **v** ^∗^. Similar to the approach we have employed for P-ROOM, we can convert this optimization problem (10) to a single-level MIP. We note that the single-level MIP equivalent of the pessimistic formulation P-OptKnock is indeed similar to the work in [26] when the tolerance level *ε* is chosen as 0. However, we emphasize that our proposed pessimistic framework is more general and can be extended to different bi-level strain optimization formulations that employ various cell-survival models.

## Results and discussion

In this section, we first evaluate the knockout solutions by optimistic bi-level optimization methods on a core *E. coli* metabolic network [27] when the outer- and inner-level decision makers are not cooperative. We then test our robust strain optimization methods, namely P-ROOM and P-OptKnock, derived in the “Methods” section, on the core network and a large *E. coli* metabolic network, iAF1260 [22], for overproduction of Succinate. The models are optimized using the MILP solver CPLEX 12.6.3 [28].

### Succinate overproduction on AntCore metabolic network

We derive knockout solutions for a core *E. coli* metabolic network model [27] with 74 chemicals and 75 reactions for Succinate overproduction. The network model is from the OptKnock package [17], in which the glucose uptake is set at a fixed value of 100mmol/gDW.hr and the minimum biomass is set as 5 mmol/gDW.hr. Since the glucose-uptake rate is fixed, the Succinate yield is equivalent to the corresponding flux value considering steady-state stoichiometry constraint. The wild-type flux distribution is computed by maximizing the biomass in the FBA framework. In this set of experiments, we set the allowable knockout numbers *K*=3, 4, and 5. All the reported experiments are based on the aerobic condition.

We first evaluate the robustness of two optimistic bi-level programs OptKnock (1) and ROOM (2) by computing the performance of least favorable Succinate overproduction under uncertainty for the derived knockouts **z** ^∗^ by solving (1) and (2). As discussed in the “Methods” section, we introduce a parameter *ε* as the model tolerance level to capture how faithful the inner-level mutant survival model is and whether the mutants will cooperate with the outer-level overproduction objectives. By allowing the gap *ε* between the realistic responses and the optimal inner-level objective function value, the increase in *ε* reflects the higher model tolerance by approximating the inner-level model uncertainty and non-cooperativeness. The pessimistic Succinate overproduction rate evaluations of optimistic knockout solutions are derived by finding the worst-case solutions of reaction flux values in *ε*-approximation sets.

**Fig. 2:**
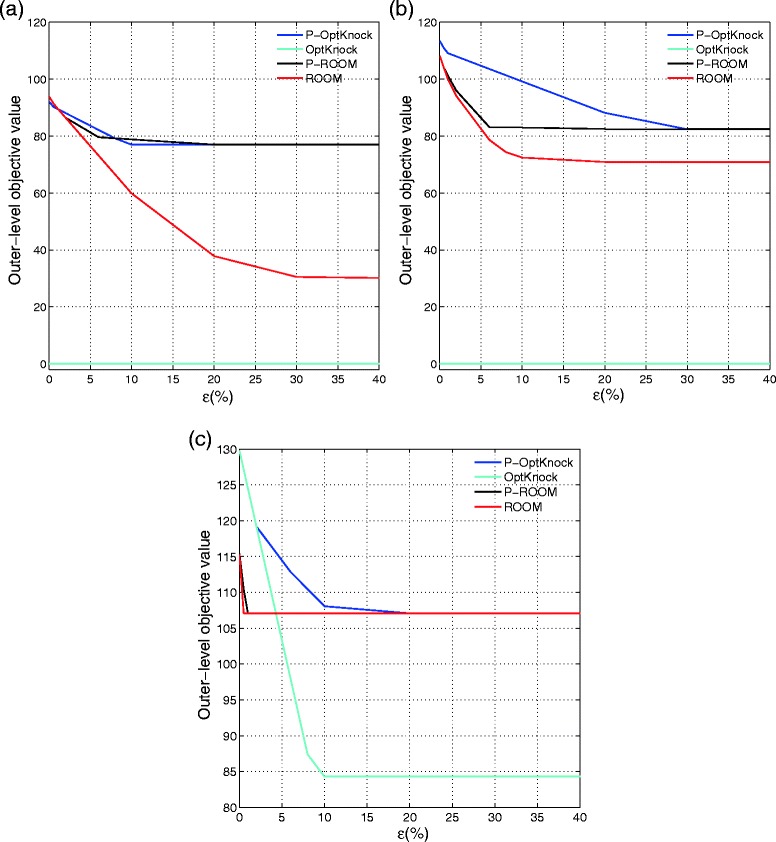
Pessimistic Models and Evaluation. The outer-level objective values of P-ROOM and P-OptKnock for **a**) *K*=3, **b**) *K*=4, and **c**) *K*=5 plotted with increasing *ε* for the core *E. coli* metabolic network. The evaluation Succinate rates of the optimistic models, ROOM and OptKnock, are also given as *red* and *cyan solid lines*, respectively. (Color-coded as *Blue*: P-OptKnock, *Black*: P-ROOM)

The computed evaluation values for different *ε* values (0≤*ε*≤0.4) are plotted in cyan and red lines in Fig. 2 with different numbers of allowed knockouts. It is clear that with higher model uncertainty (larger *ε* values), both ROOM and OptKnock strategies lead to smaller Succinate overproduction compared to the predicted optimistic overproduction rates. We also note that the Succinate rates drop very quickly even with small *ε*, which implies that these optimistic knockout strategies are not robust. Finally, when the inner-level mutant survival models are not as faithful as desired, the derived knockout strategies may not be effective at all. For example, when *K*=3 and 4, the knockouts suggested by OptKnock are too “optimistic” as the corresponding evaluation Succinate rates go to 0 as shown in Fig. 2a and b. Compared with the results from ROOM, this validates that the minimum flux change model may be a better mutant cell survival model than the maximum biomass growth model adopted in OptKnock. These experiments bring out that the cellular and engineering objectives may behave in opposite directions and the optimistic knockout strategies may not work in practice.

With the same *ε*-approximation model uncertainty, we now compare the corresponding derived knockout strategies based on our proposed pessimistic models: P-OptKnock and P-ROOM. Figure 2 illustrates the *ε*-optimal Succinate overproduction rates of the derived knockout solutions by P-OptKnock and P-ROOM in blue and black lines, respectively. Obviously, our pessimistic strategies derive more robust solutions, for which the worst-case or least favorable Succinate rates for different *ε* values are consistently on top of the rates from optimistic strategies. The least favorable Succinate rates decrease monotonically with increasing *ε* corresponding to higher model uncertainty. More interestingly, as *ε* increases, both P-OptKnock and P-ROOM converge to the stable Succinate overproduction rates. In fact, they derive the same knockout reactions as provided in Table 1. This clearly shows that our pessimistic formulations can produce consistent and robust mutant strains with respect to the inner-level mutant survival model uncertainty. As a side note, when *K*=5, the pessimistic Succinate values produced by P-ROOM and its optimistic counterpart are almost the same for different tolerance levels, probably because the surviving mutants are more restricted with the increasing number of knockout reactions. This also shows that the minimum flux change is a better mutant survival model.

**Table 1:** Knockout strains derived by pessimistic and optimistic models on the core *E.coli* metabolic network

	*K*	Knockouts	Succinate production
P-ROOM	3	6pg →ru5p + co2 + nadph, oac + accoa →cit, ac →ac(ext)	76.97
	4	g6p →6pg + nadph, oac + accoa →cit, suc ⇔fum + fadh2, ac →ac(ext)	82.36
	5	g6p →6pg + nadph, mal →pyr + co2 + nadph, 3pg + glu →ser + akg + nadh, fadh2 + 0.5o2 →2atp, nadh ⇔nadph	107.07
P-OptKnock	3	6pg →ru5p + co2 + nadph, oac + accoa → cit, ac →ac(ext)	76.97
	4	g6p →6pg + nadph, oac + accoa →cit, suc ⇔fum + fadh2, ac →ac(ext)	82.36
	5	g6p →6pg + nadph, mal →pyr + co2 + nadph, 3pg + glu →ser + akg + nadh, fadh2 + 0.5o2 →2atp, nadh ⇔nadph	107.07
ROOM	3	dhap ⇔gap, g6p →6pg + nadph, fadh2 + 0.5o2 →2atp	93.92
	4	dhap ⇔gap, g6p →6pg + nadph, fadh2 + 0.5o2 →2atp, glyc(ext) →	108.22
	5	g6p →6pg + nadph, mal →pyr + co2 + nadph, 3pg + glu →ser + akg + nadh, fadh2 + 0.5o2 →2atp, nadh ⇔nadph	115.58
OptKnock	3	g6p →6pg + nadph, mal →pyr + co2 + nadph, nadh ⇔nadph	110.179
	4	g6p →6pg + nadph, mal →pyr + co2 + nadph, 3pg + glu →ser + akg + nadh, nadh ⇔ nadph	123.314
	5	g6p →6pg + nadph, 3pg + glu →ser + akg + nadh, nadh ⇔nadph, glyc ⇔glyc(ext), ac(ext) →	129.786

In Table 1, the succinate overproduction rates are given by the pessimistic formulations P-ROOM and P-OptKnock for the tolerance values *ε* in which both models became stable (*ε*=0.4) as shown in Fig. 2. Although the pessimistic models provide different Succinate values for different *ε* values in Fig. 2, the knockout strategies and the Succinate values for both P-ROOM and P-OptKnock are identical when they reach stability. When *K*=3, one of the knockouts suggested by P-ROOM and P-OptKnock involves competing byproduct metabolism pathways for succinate such as 6-Phospho-D-gluconate (6pg) and Ribulose 5-phosphate (ru5p). With *K*=4, the reaction decomposing succinate (suc) is also eliminated. As more knockouts are allowed, the resulting succinate production can be further increased. We should also note that the removal of an important reaction for Trans-hyrogenation (nadh ⇔nadph) may cause significant reduction in biomass flux value. For the knockout strategies suggested by ROOM and OptKnock, when assuming the optimistic environment, the succinate rates are larger than the values suggested by pessimistic knockouts as expected since optimistic formulation provides an upper bound for the pessimistic formulation. From all the experiments with different *K*’s, we demonstrate that, when we formulate the mutant optimization problem as pessimistic optimization by incorporating the model uncertainty, both P-OptKnock and P-ROOM can suggest consistent and robust knockouts, making the mutant strain optimization problem more reliable, accurate, and independent from the choice of inner-level surrogate functions for mutant cell objectives.

### Succinate overproduction on iAF1260 network

We have shown that the derived knockout strategies based on optimistic methods may not be robust and often “over-optimistic” by the experiments on the core *E. coli* metabolic network, while our proposed methods P-ROOM and P-OptKnock based on pessimistic optimization for mutant strain design have achieved robust and more practical knockout strategies. We further test P-ROOM and P-OptKnock to derive mutant strains for the overproduction of Succinate on a large *E. coli* metabolic network model, iAF1260 [22], which has 1658 metabolites and 2936 reactions including the pseudo reactions required for the computational model, also from the OptKnock package [17]. The glucose uptake rate is fixed at 100 mmol/gDW.hr, and the minimum biomass value is also set to 5 mmol/gDW.hr. The reported experiments are based on the anaerobic environment. We allow *K*=3 knockout reactions on this large network.

**Fig. 3:**
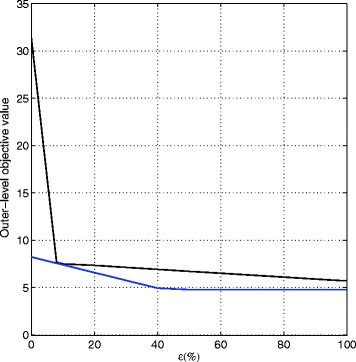
Pessimistic Models. The outer-level objective values of P-ROOM and P-OptKnock for *K*=3 plotted as *solid lines* with increasing *ε* for a large-scale iAF1260 *E. coli* metabolic network. (Color-coded as *Blue*: P-OptKnock, *Black*: P-ROOM)

Figure 3 provides the *ε*-optimal Succinate flux rates, which are the outer-level objective function values based on two pessimistic models of P-ROOM and P-OptKnock at different *ε* values. When there is no model uncertainty (*ε*=0), P-ROOM clearly provides higher Succinate rate than the value P-OptKnock suggests. This again suggests that modeling mutant cell survival by minimization of flux changes plays a role in favor of engineering objectives with improved targeted productions. At higher *ε* values, P-ROOM and P-OptKnock reach stability, and they derive the same knockout reactions as provided in Table 2 as we also observed in the experiments on the *E. coli* core metabolic network. If we consider the fact that the inner-level cell survival models are approximate to the real-world systems, incorporating this model uncertainty with *ε*-approximation in our formulated pessimistic mutant strain optimization methods, P-ROOM and P-OptKnock, has a key role to achieve robust knockout solutions that are less affected by these approximate cell survival models.

**Table 2:** Knockout strains derived by pessimistic and optimistic models on the iAF1260 *E.coli* metabolic network

	Knockouts		min v_succinate_	max v_succinate_
ROOM	-		0	0
FBA	-		0	0.372
P-ROOM	∙ mlthf + nadp ⇔methf + nadph	*ε*=0	8.09	8.09
	∙ coa + pyr →accoa + for	*ε*=1	5.69	40.08
	∙ q8 + succ →fum + q8h2			
P-OptKnock	∙ mlthf + nadp ⇔methf + nadph	*ε*=0	8.23	8.23
	∙ coa + pyr →accoa + for	*ε*=1	4.78	80.52
	∙ q8 + succ →fum + q8h2			
ROOM	∙ g6p + nadp ⇔6pgl + h + nadph	*ε*=0	31.36	31.36
	∙ h2o + pser-L →pi + ser-L	*ε*=1	0	76.143
	∙ q8 + succ →fum + q8h2			
OptKnock	∙ mlthf + nadp ⇔methf + nadph	*ε*=0	8.23	8.23
	∙ coa + pyr →accoa + for	*ε*=1	4.78	80.52
	∙ q8 + succ →fum + q8h2			

Table 2 provides the predicted knockouts by P-ROOM and P-OptKnock when *K*=3. The minimum and maximum Succinate flux rates are also given for the mutant and also wild-type strains. The Succinate flux rates of pessimistic models are given for two different *ε* values: *ε*=1 denoting the highest tolerance level for the inner-level model uncertainty, and *ε*=0 denoting 0 tolerance level. In Table 2, both P-ROOM and P-OptKnock predict three identical knockouts that yield a minimum production rate of the Succinate that is higher than the predicted production rate in the wild-type strains. It validates that our pessimistic mutant strain optimization methods guarantee a higher Succinate production rate than that of wild-type strains even in the worst-case scenario. Furthermore, minimum and maximum Succinate rates given the predicted knockouts define a larger range for the OptKnock model, which may indicate the inner-level mutant cell survival model by biomass maximization is less faithful than ROOM’s minimum flux change model.

We have also included the derived knockouts by ROOM and OptKnock and corresponding minimum and maximum succinate rates in Table 2. The mutant strain with the knockouts obtained by ROOM produces 0 minimal succinate flux value with the highest inner-level model uncertainty. However, P-ROOM succeeds in getting higher production rates even when *ε*=1. The minimal succinate rate when *ε*=0 in ROOM is greater than the minimal succinate rate for P-ROOM which is 8.09. This is because the reported pessimistic knockouts are identified when the model uncertainty is taken as *ε*=1. We note that the mutant with the pessimistic knockouts identified by P-ROOM when *ε*=0 actually produces the minimal succinate rate as 31.36. The knockouts captured by OptKnock are same as the ones identified by pessimistic formulations, leading to the same mutant with the same succinate production rates as P-OptKnock. Pessimistic formulations identified the succinate dehydrogenase reaction (SUCDi) as one of the suggested knockouts, which directly consumes succinate (succ), and the reaction pyruvate formate lyase (PFL). These have been reported as effective knockout strategies in [17, 29].

As demonstrated by the results from both the small core *E. coli* metabolic network and large iAF1260 network, our pessimistic formulations for strain optimization produce robust and stable knockout solution strategies under model uncertainty. Especially when comparing the results from P-ROOM and P-OptKnock, both models derive the same stable knockout solutions regardless of the inner-level surrogate functions for mutant cell survival. Such consistency is critical when considering the translation of computationally derived results into in vivo experiments in practical metabolic engineering.

## Conclusions

In this paper, we have proposed a new pessimistic optimization framework to identify optimal knockout strategies for maximum targeted bio-production under model uncertainty. We specifically have investigated two cell survival models and presented two corresponding pessimistic models to derive robust knockout reactions to achieve maximum biochemical overproduction: (1) P-ROOM under the minimum flux change condition; and (2) P-OptKnock under the maximum biomass condition. The experiments on both the core *E. coli* metabolic network [27] and large-scale iAF1260 network [22] have demonstrated that the pessimistic models for strain optimization derive robust and stable metabolic strain perturbation strategies through genome-scale steady-state stoichiometric modeling. Formulating the strain optimization problem based on pessimistic optimization considering the model uncertainty leads to consistent and reliable solutions regardless of the inner-level mutant survival models, which can provide high confidence for future in vivo experimental design of promising microbial mutants that benefit the human society.
